# Tuning thermal and graphitization behaviors of lignin *via* complexation with transition metal ions for the synthesis of multilayer graphene-based materials†

**DOI:** 10.1039/d3ra05881f

**Published:** 2024-03-04

**Authors:** Qiangu Yan, Hanwen Zhang, Timothy Ketelboeter, Yucheng Peng, Caixia Wan, Zhiyong Cai

**Affiliations:** a Forest Products Lab, USDA Forest Service One Gifford Pinchot Drive Madison WI 53726 USA zhiyong.cai@usda.gov +1 608 231 9446; b Department of Chemical and Biomedical Engineering, University of Missouri 1406 East Rollins Street Columbia MO 65211 USA wanca@missouri.edu +1 573 884 7882; c College of Forestry, Wildlife and Environment, Auburn University 602 Duncan Dr Auburn AL 36849 USA yzp0027@auburn.edu +1 334 844 1089

## Abstract

Thermal conversion of kraft lignin, an abundant renewable aromatic substrate, into advanced carbon materials including graphitic carbon and multilayer/turbostratic graphene has recently attracted great interest. Our innovative catalytic upgrading approach integrated with molecular cracking and welding (MCW) enables mass production of lignin-derived multilayer graphene-based materials. To understand the critical role of metal catalysts in the synthesis of multilayer graphene, this study was focused on investigating the effects of transition metals (*i.e.*, molybdenum (Mo), nickel (Ni), copper (Cu), and iron (Fe)) on thermal and graphitization behaviors of lignin. During the preparation of metal-lignin (M-lignin) complexes, Fenton-like reactions were observed with the formation of Fe- and Cu-lignin complexes, while Ni ions strongly interacted with oxygen-containing surface functional groups of lignin and Mo oxyanions weakly interacted with lignin through ionic bonding. Different chelation mechanisms of transition metal ions with lignin influenced the stabilization, graphitization, and MCW steps involved in thermal upgrading. The M-lignin complex behaviors in each of the three steps were characterized. It was found that multilayer graphene-based materials with nanoplatelets can be obtained from the Fe-lignin complex *via* MCW operation at 1000 °C under methane (CH_4_). Raman spectra indicated that Fe- and Ni-lignin complexes experienced a higher degree of graphitization than Cu- and Mo-lignin complexes during thermal treatment.

## Abstract 2

Transition metals affect thermal stabilization and graphitization of kraft lignin and further determine morphology and graphitic structures of multilayer graphene-based materials.

## Graphical abstract

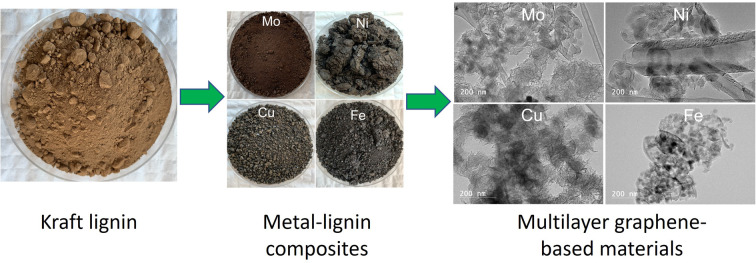

## 1. Introduction

Graphene is a two-dimensional sheet material made of pure carbon atoms, with each carbon atom involved in three sigma (σ) bonds and one pi (π) bond.^1^ Excellent in-plane properties, including high electrical (∼10^4^ Ω^−1^ cm^−1^) and thermal conductivity (∼3000 W m^−1^ K^−1^) as well as high mechanical stiffness of hexagonal network (∼1060 GPa)^1^ have attracted significant interest from various academic and industrial sectors (*e.g.*, medical devices,^2^ sensors,^3^ fuel cells,^4^ batteries,^5^ display materials,^6^ packaging^7,8^). The demand for sustainable development of graphene materials has driven those sectors to search for renewable resources to replace petroleum sources as precursors. To this end, lignocellulosic biomass presents an abundant and low-cost source for graphene and other advanced carbon materials. Especially, lignin appears to be the most suitable component in lignocellulosic matrix for graphene formation due to abundant aromatic subunits and high carbon content. However, research efforts in manufacturing graphene materials from lignocellulosic biomass and lignin are still limited.

Lignin is produced at large quantities (>50 million metric tons) annually as a waste by-product especially from wood delignification process in pulp and paper industry.^9,10^ Kraft lignin accounts for 90% technical lignin worldwide since kraft process is prevailing in pulping mills.^11,12^ Despite great potential of lignin for aromatic chemicals and materials, technical lignin still remains underutilized, with only up to 2% exploited for commercial use, and the remainder is typically combusted as low quality solid fuels.^13^ Research efforts in promoting lignin valorization especially toward graphitic materials especially with graphene domains would open a new avenue to value-added upgrading of technical lignin. For example, lignin-derived carbon fibers,^14,15^ graphene,^16–18^ and carbon foams^19^ have exhibited intriguing properties and great potential for multifunctional applications. Recently we have developed a novel process for production of multilayer graphene-based materials at tens of gram scale, which is a great step forward toward large-scale operation practices considering time/energy/labor constrains in current graphene manufacturing (*e.g.*, chemical vapor deposition, Hummers' method). The process involves four main steps, including the formation of transition metal-lignin (M-lignin) *via* co-precipitation, stabilization of M-lignin composites by mild thermal treatment (≤300 °C), catalytic graphitization of M-lignin composites to graphene-encapsulated metal (M@G) structures, and separation of graphene shell from M@G structure by a molecular cracking and welding (MCW) operation. Moreover, the MCW operation enables additional tailoring of graphene properties while facilitating the separation of multilayer graphene shells from metal particles.^20^ This novel process is scalable and proved to be highly effective for transforming kraft lignin into multilayer graphene-based materials.

Our prior research studied the significant influence of various metal catalysts on the thermal graphitization of kraft lignin especially in relation to carbon solubility and propensity for metal carbide formation.^16^ The MCW operation was found to play an important role in the graphene manufacturing process as different operation conditions of this step can result in graphene materials with different forms, such as nanoplatelets, sheets, and fluffy agglomerates.^18,20^ For example, the temperature has a profound effect on the degree of graphitization, while atmosphere (gas medium) for MCW can influence graphitic structures, morphologies, and yields. However, how the structure and properties of M@G materials are altered by different metal catalysts during the MCW process remains unclear. To gain a deeper understanding of the transformation mechanisms and fine tune the process toward mass production, it is important to study how different transition metals play their roles in MCW operation.

The main objective of this study was to investigate the effects of transition metals on thermal and graphitization behaviors of lignin. Four transition metals, including iron (Fe), copper (Cu), nickel (Ni), and molybdenum (Mo), were studied using respective salts and compared for their effects on the yields, morphology, and main characteristics of the lignin-derived graphitization products. This research offered new insights into lignin graphitization.

## 2. Experimental section

### 2.1. Materials

Iron(iii) nitrate nonahydrate (Fe(NO_3_)_3_·9H_2_O, >97% purity), copper(ii) nitrate tetrahydrate (Cu(NO_3_)_2_·4H_2_O, >98% purity), nickel(ii) nitrate hexahydrate (Ni(NO_3_)_2_·6H_2_O, >98% purity), and ammonium molybdate tetrahydrate ((NH_4_)_6_Mo_7_O_24_·4H_2_O, >97% purity). Tetrahydrofuran (THF) were purchased from Sigma-Aldrich (St. Louis, MO, USA). Kraft lignin (BioChoice lignin) was supplied by Domtar Inc (Plymouth, NC, USA).^21^

### 2.2. Preparation of M-lignin complexes

The M-lignin composites based on different transition metals were prepared by the co-precipitation method as described in our prior study.^21^ Lignin solution was prepared by dissolving 100 g of kraft lignin in 150 mL of THF with continuous stirring for 60 min at 50 °C. The ratios of moles of Fe, Ni, Cu, and Mo to mass of lignin were 0.20, 0.16, 0.31, and 0.26 mmol g^−1^, respectively. The as-prepared lignin solution and metal salts were mixed with continuous stirring for 30 min at 50 °C. The mixtures were then dried at room temperature in the fume hood for about one week until THF in M-lignin composites was completely evaporated. Finally, the dry solids (M-lignin composites) were collected and denoted as M-lignin composites with M referred to respective transition metals, *i.e.*, Fe-lignin, Cu-lignin, Ni-lignin, and Mo-lignin.

### 2.3. Stabilization of M-lignin composites

The as-prepared M-lignin composites were subjected to thermal treatment for improved stability. In brief, M-lignin composite was loaded to a muffle furnace under nitrogen flow (100 mL min^−1^) for 30 min. The M-lignin composite was then treated in the muffle furnace by increasing the temperature to 300 °C at a heating rate of 2.5 °C min^−1^, then holding at 300 °C for 30 min, and finally cooling to room temperature under nitrogen flow (100 mL min^−1^). Thermal stabilization process was also studied by performing thermogravimetric analysis (TGA) of the M-lignin 300 °C using TGA-50H analyzer (Shimadzu, Columbia, Maryland, USA). Kraft lignin only was used as a control. The analysis was carried out at the temperature range of 25–300 °C with a heating rate of 5 °C min^−1^. In each run, 10 mg of the samples was used under argon at a flow rate of 50 mL min^−1^. The tests were done in triplicate for each sample. DSC measurement was performed using a differential scanning calorimeter Q2000 (TA Instrument, New Castle, DE, USA) coupled with a cooling system down to −80 °C. Approximately 10 mg of sample was loaded to an air-tight aluminum pan and heated with a heating rate of 5 °C min^−1^ from 25 to 300 °C under nitrogen flow (50 mL min^−1^). Thermal response of the sample was characterized.

The main gaseous products released from the stabilization process of the M-lignin material were characterized by an online Hiden QGA quantitative gas analysis system (Hiden Analytical, Livonia, Michigan, USA). In brief, 5 g of M-lignin composite was loaded to a bench-scale fixed bed tubular reactor made of 1′′ OD stainless steel. The tubular reactor was heated with a heating rate of 5 °C min^−1^ from 25 to 300 °C and then kept at 300 °C for 5 min. During the experiment, the sample temperatures were measured using a type K thermocouple inserted in the center of the sample bed. As the reaction temperature increased from 25 to 300 °C, the gaseous products with mass spectra of 2, 15, 28, 34, 44, 78, and 94 (*m*/*z*) were identified and the evolution profiles of main components including H_2_, CH_4_, CO, CO_2_, and NO_2_ were quantified.

### 2.4. Catalytic graphitization of M-lignin composites

For catalytic graphitization, thermally stabilized M-lignin composites (∼50 g) was first ball milled at 1000 rpm for 10 min and then loaded to the center of a 2′′ outer diameter (O.D.) ceramic tube inside a furnace. Argon (99.99% purity) was introduced as a carrier gas to the tubular furnace at a flow rate of 100 mL min^−1^ for 30 min before the thermal treatment. The catalytic graphitization of the M-lignin material was performed by increasing the furnace temperature from room temperature to 1000 °C at a heating rate of 10 °C min^−1^, followed by holding at 1000 °C for 1 h. After graphitization, the furnace was cooled down to room temperature at a rate of 10 °C min^−1^ under argon flow (100 mL min^−1^), and the M@G materials in the powder form were collected.

### 2.5. MCW operation

To complete the MCW operation, ∼50 g of the above-prepared M@G material was placed in the middle of a 1′′ O.D. tubular reactor. Then, CH_4_ was used as a gas for simultaneous cracking and welding and introduced to the furnace at a flow rate of 50 mL min^−1^. The MCW operation was initiated by heating the tube furnace to 1000 °C at a heating rate of 10 °C min^−1^ and holding at the same temperature for 1 h. The system was finally cooled to room temperature at a rate of 10 °C min^−1^. The graphene-based powder samples were collected and used for characterization.

### 2.6. Characterization

The crystalline structure of the M@G materials was characterized by X-ray powder diffraction (XRD). XRD patterns were acquired on a Rigaku Ultima III X-ray Diffraction System operated at 40 kV and 44 mA using Cu-Kα radiation with a wavelength of 1.5406 Å from 20 to 80° at a scan rate of 2° s^−1^. The JADE powder diffraction analysis software (Materials Data, Inc.) was used for both qualitative and quantitative analysis of polycrystalline powder materials. The ultrastructures of M@G samples were examined using a JEOL JEM-100CV II transmission electron microscope (TEM) operated at an accelerating voltage of 200 kV. The yields of the M@G samples were calculated as the mass difference between M-lignin composite and M@G powder.

The yields of the products resulting from MCW were characterized through a temperature-programmed oxidation process using TGA-50H analyzer ((Shimadzu, Columbia, Maryland, USA). In each run, 20 mg of M@G sample was thermally treated by heating the temperature from room temperature to 800 °C at a heating rate of 10 °C min^−1^ under air flow (100 mL min^−1^). The mass loss characterized by TGA was recorded as the mass loss of amorphous carbon in the M@G sample after MCW. The mass of residues after the TGA was considered the mass of multilayer graphene-based materials, which was used for calculating the yields. XRD and TEM used for characterizing the products resulting from MCW were same as described above. Morphology of the materials obtained through the MCW operation was investigated using a scanning electron microscope (JEOL, Peabody, MA, USA) operated at 10 kV. All the samples were coated with 10 nm of platinum before being loaded into the SEM vacuum chamber. Raman spectra were acquired on a Jobin-Yvon microspectrometer (Edison, NJ, USA) equipped with an excitation laser source emitting at 514 nm and an incident power around 1 mW on a thin surface. Twenty spectra were acquired for each sample, and deconvolution of the spectra was performed with the assumption of mixed Gaussian/Lorentzian peaks describing both the main D and G bands and two minor bands. The D, G, and 2D peaks in the Raman spectra were fitted with Lorentz functions. The ratios of *A*_D_/*A*_G_, which are associated with the graphitic degree, were calculated using the integrated areas of the D and G peaks.

## 3. Results and discussion

### 3.1. Preparation of M-lignin complexes

A critical step in this work was to uniformly disperse and distribute transition metals in the matrix of kraft lignin *via* solution co-precipitation to facilitate effective catalytic graphitization. Upon mixing lignin solution and metal salt solutions, the metal ions migrated to the surface of the lignin and became chelated with the O- and S-containing surface functional groups, forming the M-lignin complexes. The complexes precipitated at a critical concentration due to nucleation and particle size growth. In the process of M-lignin complex formation, there were several key factors, including types of transition metals, complex ion concentration, lignin-dissolving solvents, pH, temperature, all influencing M-lignin complex characteristics (*e.g.*, morphology, structure, uniformity, metal oxidation states).^22^

When different transition metals (*i.e.*, Fe, Cu, Ni, and Mo) were used, co-precipitation phenomena varied. The solution temperatures of all the metals except Mo drastically increased during the first 25 s of mixing and tended to level off or slightly decrease (Fig. S1a†). The peak temperatures for the three transition metals (Fe, Cu, and Ni) were approximately 77, 72, and 56 °C, respectively, while the solution temperature for Mo remained almost constant at 50 °C throughout the mixing. Temperature increase can be triggered based on several mechanisms involved in the complexation of metal ions and lignin. One could be that upon mixing, metal ions may diffuse or transfer to different lignin surface areas, forming a M-lignin complex with coordinated chemical bonds between metal ions and O-/S- containing surface functional groups of lignin. Another mechanism could be energy changes associated with M-lignin particle size growth and precipitation. Exothermic reactions, such as Fenton-like reaction in the case of ferric ion reacting with functional groups in lignin, can also contribute to increased temperature.^23,24^

Lignin consists of various functional groups (*e.g.*, hydroxyl, carboxyl, carbonyl, phenolic, ether, lactone). These functional groups serve as ligands to trap metal ions by coordinating bonding for the formation of M-lignin complexes. Different transition metal ions possessing various oxidation states and electron configurations demonstrated significantly different interactions with kraft lignin during complexation and co-precipitation. For example, Fe^3+^ can chelate with abundant lignin functional groups, facilitating better dispersion of metal ions in lignin. Gaseous byproducts identified as CO_2_ and nitrogen oxide (NO_2_) were released because of Fenton-like reactions (Fig. S1b and c†). Generation of both CO_2_ and NO_2_ occurred at the beginning of mixing process, and the concentration of both gases rapidly increased with the continued mixing and then declined. The Fe^3+^-lignin solution was initially clear, then foamed due to the release of gaseous byproducts, and finally the particles with dark grey color precipitated out along with the collapse of foam. In contrast, Cu-lignin complex was formed in a similar way likely due to Fenton-like reaction involved in Cu-catalyzed reactions (Fig. S1 and S2†), but showed a different color, lower peak temperature, and lower gaseous byproduct concentrations. The Ni-lignin complex formation process differed from the Fe- and Cu-based reaction systems as it showed a much lower solution peak temperature. In addition to chelation, Ni^+^ significantly modified the molecular structure of lignin by forming agglomerates among lignin molecules, leading to condensed materials.^25^ These agglomerates of Ni-lignin appeared to be large, grey lumps as shown in Fig. S2.† Highly viscous syrup-like solution was formed with no generation of gaseous byproducts (Fig. S1b and c†).

The transition metal Mo(vi) in the ammonium heptamolybdate solution exists in the oxyanion form (Mo_7_O_24_^6−^) and is unlikely to chelate onto the lignin surface rich in negatively charged oxygen-containing functional groups. In this case, ammonium cations (NH_4_^+^) might be attached to the lignin surface and the interactions between lignin and Mo would be through the ionic bonding between NH_4_^+^ and Mo_7_O_24_^6−^. Thus, neither solution temperature changed nor gaseous products generation occurred while mixing the ammonium heptamolybdate tetrahydrate and lignin solutions (Fig. S1†). Not surprisingly, the Mo-lignin complex powder had a similar color to kraft lignin (Fig. S2†) as Mo in the oxyanion form should be only physically dispersed onto the lignin surfaces.

### 3.2. Stabilization of M-lignin complexes

**Fig. 1:**
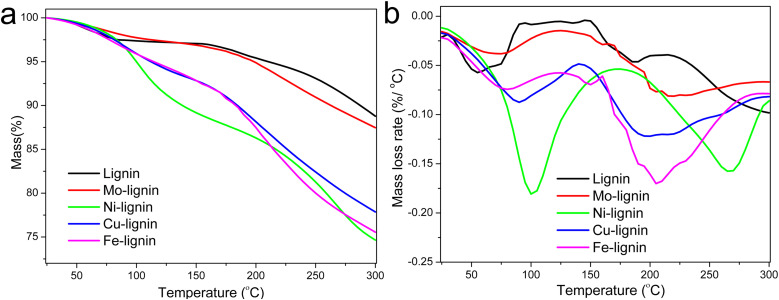
TGA (a) and DTG (b) curves of lignin and M-lignin complexes during the stabilization process.

The stabilization of M-lignin is a crucial pre-decomposition step that facilitates the reaction at relatively low temperatures (up to 300 °C) before the catalytic graphitization process takes place. Without such a step, the M-lignin complexes could experience a thermal runaway event during the heating process, as suggested in our prior study concerning thermal stability of Fe-lignin composites.^26^ The mass loss of lignin and M-lignin complexes with increasing temperature during the thermal stabilization process was characterized by TGA. The mass loss of kraft lignin can be characterized by three steps in the temperature range of 25–300 °C (Fig. 1a), including the 1st step for water evaporation (25 to ∼100 °C), the 2nd step for loss of chemically bonded water from 150–220 °C, and the 3rd mass loss step at ∼220 °C for the thermal degradation of lignin. The residual mass of kraft lignin at 300 °C was around 88.5 wt%.

M-lignin complexes showed different mass loss profiles from 25–300 °C compared to kraft lignin (Fig. 1a). Collectively, M-lignin complexes showed two main steps of mass loss, with the 1st one for the loss of adsorbed water at 25–100 °C and the 2nd step for thermal degradation of lignin. However, the release of coordinated water from the complexes was also observed and thus shifted the DTG peak for mass loss to a higher temperature compared to that of kraft lignin at 60 °C (Fig. 1b). At the 2nd step, the peak temperature of the lignin degradation shifted to about 30–100 °C higher than kraft lignin due to the catalytic degradation of lignin especially involved in the cleavage of ether and C–C bonds by transition metals in the complexes.^26^ The final residual mass at 300 °C for the Mo-, Cu-, Ni-, and Fe-lignin complexes was 87.0, 75.5, 74.5, and 78.0 wt%, respectively. The TGA results indicated that different transition metals significantly affected the thermal stabilization process of M-lignin complexes at the treatment temperature range of 25–300 °C. The thermal behavior analysis also demonstrated that Fe and Cu had similar impacts on shifting the thermal degradation temperature of lignin, which was consistent with observations during the M-lignin complex preparation process.

**Fig. 2:**
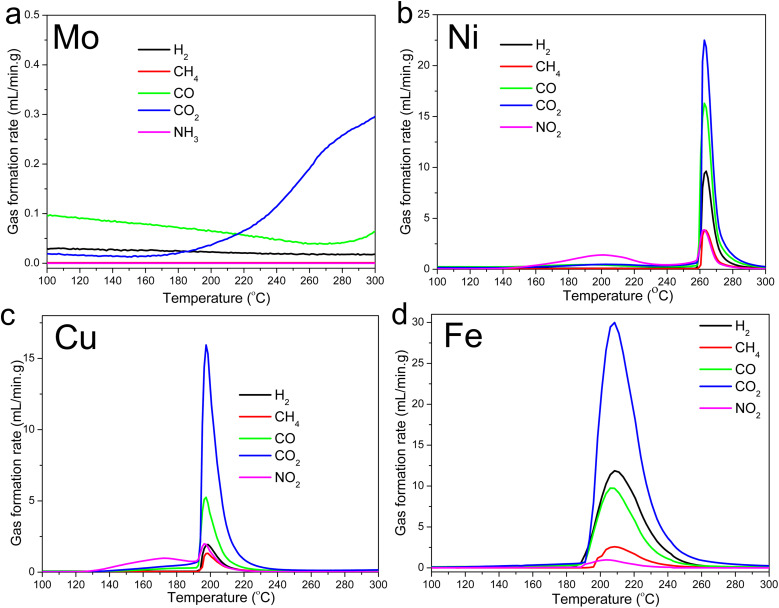
Gas evolution profiles during the M-lignin complex stabilization process: (a) Mo-lignin, (b) Ni-lignin, (c) Cu-lignin, and (d) Fe-lignin.

The gas evolution profiles for the Ni-, Cu-, and Fe-lignin complexes showed that big amounts of gases were generated during the stabilization process (Fig. 2). The Ni-, Cu-, and Fe-lignin complexes had their maximum gas generation rates observed at 262, 200, and 209 °C, respectively (Fig. 2b–d), which overlapped with the temperatures for their maximum mass loss rate identified in the DTG curves (Fig. 1b). The transition metals in the Ni-, Cu-, and Fe-lignin complexes promoted the generation of gases mainly from the thermal decomposition of lignin.^26^ The maximum CO_2_ generation rates for the Ni-, Cu-, and Fe-lignin complexes were 22, 16, and 30 mL min^−1^-g, respectively. The gas generation rates for all the other four gases, including H_2_, CH_4_, CO, and NO_2_, also varied across the M-lignin complexes. In contrast, the gas evolution profiles of the Mo-lignin complex did not change significantly compared with that of kraft lignin because there was no ionic bonding between Mo ion and lignin.

**Fig. 3:**
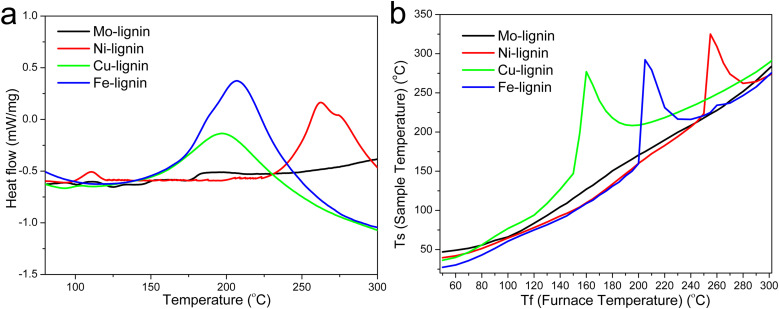
(a) DSC thermograms of the M-lignin complexes and (b) temperature profiles of the M-lignin complexes during the stabilization process.

The DSC thermograms in the temperature range of 75–300 °C for all the M-lignin complexes are shown in Fig. 3a. Different M-lignin complexes demonstrated different thermal behaviors. As the temperature increased to the thermal degradation temperature of M-lignin complexes, chemical reactions occurred, resulting in the changes in the heat flow of the materials. Under this circumstance, the exothermic peaks from the DSC measurements appeared at the temperatures matching the peak temperatures identified on the DTG curves and in the gas evolution profiles, *i.e.*, at around 262, 200, 209 °C for Fe-, Cu-, and Ni-lignin complexes, respectively (Fig. 3a, 1b and 2). These exothermic peaks resulted from the M-lignin complexes' thermal decomposition releasing heat. In contrast, the Mo-lignin complex began releasing heat at temperatures approaching the end of the DSC measurement (Fig. 3a). Lignin complexed by different transition metals showed different chemical reactions during the stabilization process, leading to varying amounts of heat released from the system. The complexes' temperatures during the stabilization process were also measured, and the data are shown in Fig. 3b. As the furnace temperature increased from 25 to 300 °C, the temperature of the M-lignin complexes changed correspondingly since no chemical reactions were occurring. However, the temperature spikes were observed during the stabilization processes of all the M-lignin complexes except Mo-lignin complex. The temperature spikes of the Cu-, Fe-, and Ni-lignin complexes were 275, 290, and 325 °C (corresponding to the furnace temperatures at 161, 208, and 258 °C), respectively. The data shown in Fig. 3b indicated that the thermal stability of the M-lignin complexes followed Mo > Ni > Fe > Cu. The transition metal complexation changed the thermal behavior of lignin, *i.e.*, decreasing its heat capacity compared to that of pristine kraft lignin.

### 3.3. Catalytic graphitization of M-lignin complexes

**Fig. 4:**
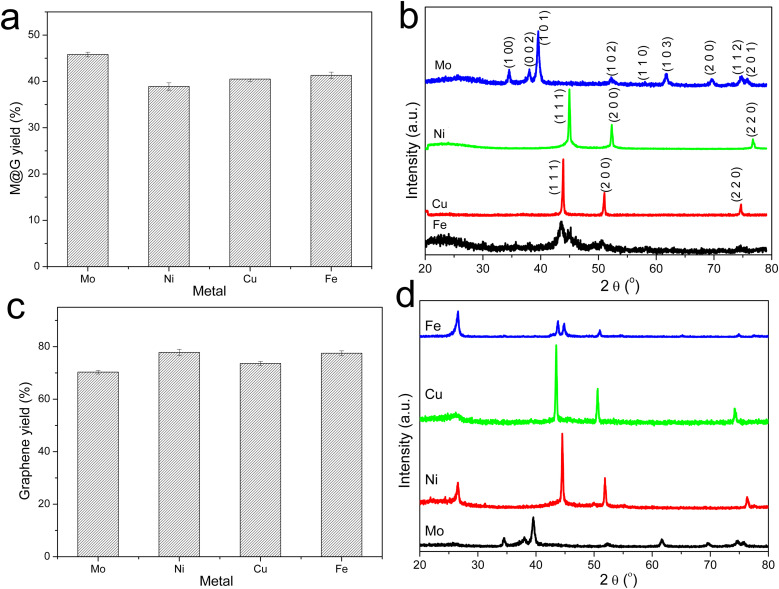
Product yields and XRD spectra. (a) Yields of M@G from the graphitization process. (b) XRD spectra of the M@G after graphitization. (c) XRD spectra of the products resulting from MCW operation. (d) Yields of the products resulting from MCW operation.

The graphitization of stabilized M-lignin complexes was performed by heating the materials from 25 to 1000 °C at a heating rate of 10 °C min^−1^. The thermal process resulted in the M@G particles similar to that reported in our prior study.^27^ The M@G yields varied across the M-lignin complexes due to different stabilization behaviors (Fig. 4a). The Mo-lignin complex had the highest yield (49.8 wt%) of M@G, followed by Fe (42.6 wt%), Cu (41.8 wt%), and Ni (38.9 wt%). Prior studies also reported the formation of core–shell structure of metal carbide with ordered carbon materials as a shell surrounding a core of metal nanoparticles.^16,18^ Two main theories can explain the mechanisms of forming ordered carbon structures. The dissolution–precipitation theory proposes that the carbon atoms in the disordered form can diffuse and dissolve into metal and/or metal carbide at elevated temperatures, followed by carbon precipitation when the system cools down due to the supersaturation of carbon dissolved in metal.^28,29^ The carbon precipitated from metal during cooling forms graphitic materials since graphite is a highly ordered carbon form with the lowest Gibbs free energy.^30,31^ The M-lignin complex preparation process uniformly dispersed and distributed the metal ions into the kraft lignin matrix, and the graphitization process then formed uniformly dispersed, ordered carbon structures surrounding the metals. During the thermal treatment of M-lignin complex,^16^ carbon is dissolved into the metals at elevated temperatures resulting in metal carbide. The controlled cooling process after graphitization facilitated the formation of ordered carbon structures. The other main theory proposes that the ordered carbon formation is the formation-decomposition process of metal carbide.^32,33^ In this theory, amorphous carbon reacts with metals at elevated temperatures to form metal carbides, then decomposes at higher temperatures to generate metal and ordered carbon in the graphite form. In either mechanism, the formation of ordered carbon possibly occurred in the graphitization process, and the temperature of the graphitization process is critical for catalytically converting lignin into graphitic materials.

**Fig. 5:**
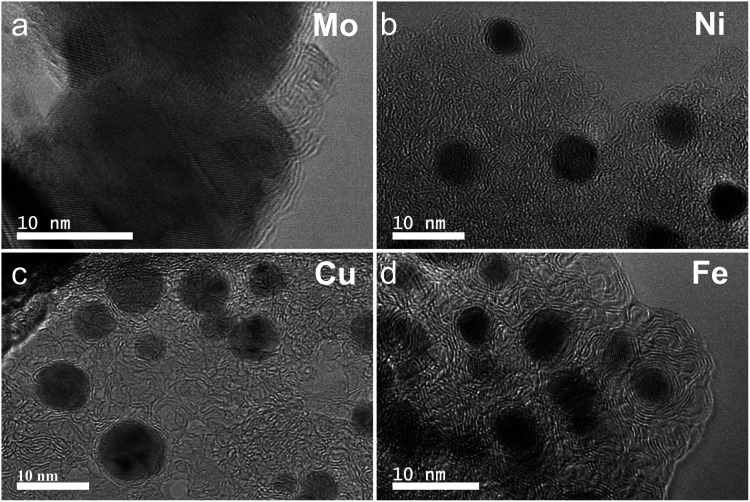
TEM images of the M-lignin complexes after graphitization: (a) Mo-lignin, (b) Ni-lignin, (c) Cu-lignin, and (d) Fe-lignin.

In the process of forming an ordered carbon structure, the metal was also converted into metal particles with different crystalline structures. The XRD spectra of the graphitized Mo-lignin complex showed the diffraction peaks at 2*θ* = 34.3°, 39.6°, 37.8°, 52.3°, 54.4°, 61.5°, 69.8°, and 74.5°, which were assigned as the crystalline planes with the Miller indices of 100, 101, 002, 102, 100, 110, 103, and 112, respectively, for the β-Mo_2_C structure (Fig. 4b). The Ni-lignin complex after graphitization showed the main diffraction peaks of 2*θ* = 44.5°, 51.8°, and 76.4°, which are the characteristic of face-centered cubic (fcc) nickel crystalline structure with the corresponding crystalline planes of 111, 200, and 220 (Fig. 4b). The main diffraction peaks for the graphitization products of the Cu-lignin complex are 2*θ* = 43.2°, 50.4°, and 74.0°, which were assigned to the crystalline planes of 111, 200, and 220 of the fcc crystals (Fig. 4b). For the Fe-lignin complex, the graphitization process generated three different crystalline structures, including α-Fe, γ-Fe, and cementite, with corresponding diffraction peaks shown in Fig. 4b.^16^ TEM images clearly showed the M@G structure with layers of graphene shell (Fig. 5). The sizes of the other three transition metal crystals are generally smaller than 10 nm, while β-Mo_2_C showed the large particle sizes (>10 nm) and lower-quality graphene layers. Compared to the physical dispersion of Mo oxyanion onto lignin, the uniform dispersion and distribution of other metal ions within the M-lignin complexes *via* direct chelation facilitated generating smaller metal nanocrystals during the graphitization process.

### 3.4. MCW operation for producing multilayer graphene-based materials

Multilayer graphene-based materials were produced *via* MCW operation using CH_4_ as a cracking and welding gas. As revealed by our prior study,^18^ the dual roles CH_4_ played are to effectively break the deposits of graphene layers from the transition metal surfaces while simultaneously welding cracked graphene layers together, endowing the materials with new properties such as morphologies. The yields of multilayer graphene-based materials from the MCW operation were determined by burning off amorphous carbon from the cracked and welded M@G materials (Fig. 4c). Ni@G and Fe@G gave the much higher yields of graphene materials (both about 77% yield), while Mo@G had the lowest yield of graphene (about 70%) due to much more amorphous carbon. XRD spectra (Fig. 4d) showed metal crystalline structures did not change after the MCW operation compared with the metal structures resulting from the graphitization process (pre-MCW).^18^ The relative intensities of the graphene peak (2*θ* = 26.5°, corresponding to the (002) plane of the graphene layer) in the XRD spectra were significantly increased for all the transition metals except Mo, indicating significant increases in multilayer graphene-based materials after the MCW operation.

**Fig. 6:**
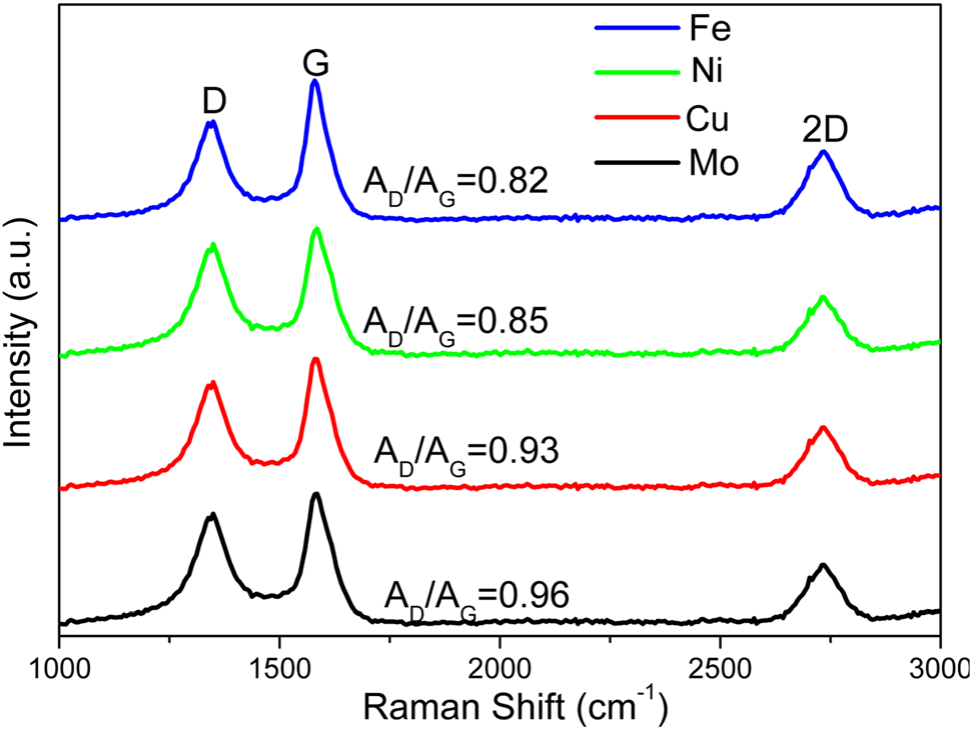
Raman spectra of the products resulting from MCW.

The Raman spectra of multilayer graphene-based materials resulting from the MCW operation are shown in Fig. 6. The *A*_D_/*A*_G_ ratios for multilayer graphene-based materials generated from the Mo-, Cu-, Ni-, and Fe-lignin complexes were 0.96, 0.93, 0.85, and 0.82, respectively. A higher *A*_D_/*A*_G_ ratio indicated a higher defect level in multilayer graphene-based materials. Therefore, the graphitization process and the MCW operation generated a higher degree of graphitization with less defect formation in the Fe- and Ni-lignin complexes than in the Cu- and Mo-lignin complexes. Compared to the Raman spectra of the M@G materials (*i.e.*, pre-MCW), the post-MCW graphene materials showed the lower *A*_D_/*A*_G_, indicating the lower defect densities in the structures. This also suggested that certain defect structures can be removed during the MCW process.

**Fig. 7:**
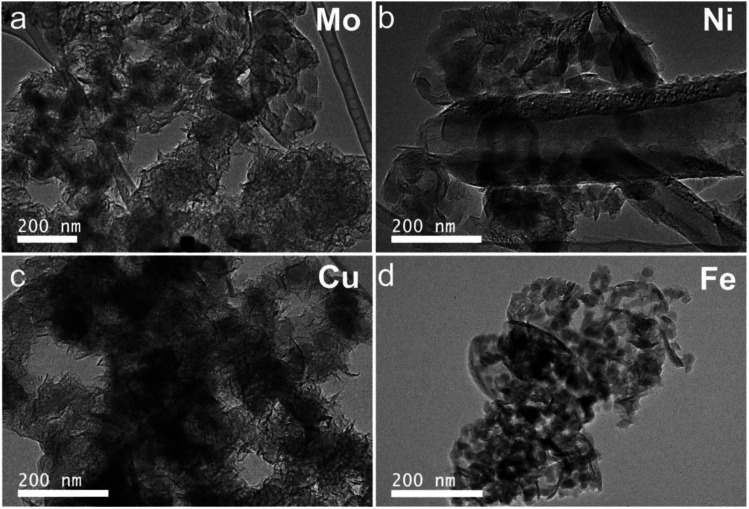
TEM images of ultrastructures and morphologies of the products resulting from MCW: (a) Mo-lignin, (b) Ni-lignin, (c) Cu-lignin, and (d) Fe-lignin.

The morphologies of graphene characterized by SEM are shown in Fig. S3.† Different morphologies were observed with post-MCW products from different M-lignin complexes. Fluffy flake shape with a connected structure were observed with the products based on both the Cu- and Mo-lignin complexes, with the smaller flakes presented in the Mo-lignin complex. The multilayer graphene-based materials based on Ni-lignin complex showed numerous particles and chips. Platelet shapes were identified in the multilayer graphene based on Fe-lignin complex. Such observations were corroborated by TEM images of the respective products (Fig. 7). These findings suggest the transition metals significantly influenced the morphologies of graphitized lignin after MCW operation.

## 4. Conclusions

Transition metals, including Fe, Cu, Ni, and Mo, were investigated for their effects on thermal and graphitization behaviors of lignin-metal complexes during the transformation of lignin into multilayer graphene-based materials. The molecular cracking and welding (MCW) operation that followed graphitization did not change the crystalline structure of the transition metals. The yields of multilayer graphene-based materials resulting from MCW reached about 78% for the Fe- and Cu-based complexes, which was 4–8% higher than that based on the other two metal-lignin complexes. Different morphologies and microstructures were observed with the multilayer graphene-based materials resulting from MCW, *i.e.*, nanoplatelets based on Fe-lignin, chips and particles based on Ni-lignin, fluffy flakes based on Mo- or Cu-lignin. Raman spectra indicated that a higher degree of graphitization was achieved with the Fe- and Ni-lignin complexes than with the Cu- and Mo-lignin complexes. Overall, Fe-lignin complex appeared to be the best in terms of the yields of multilayer graphene-based materials and well-defined graphene domains. This work revealed the transition metal-lignin complexation significantly influenced the thermal and graphitization behaviors of lignin. Higher-quality multilayer graphene-based materials with tailored morphology and properties can be obtained by designing such metal-lignin complexes.

## Conflicts of interest

There are no conflicts to declare.

## Supplementary Material

RA-014-D3RA05881F-s001
