# Supplementary material for: Tuning thermal and graphitization behaviors of lignin via complexation with transition metal ions for the synthesis of multilayer graphene-based materials
Source: RSC Adv. 2024 Mar 4;14(11):7592–600. doi: 10.1039/d3ra05881f (PMC10910854; doi:10.1039/d3ra05881f)
Supplement: RA-014-D3RA05881F-s001 [file RA-014-D3RA05881F-s001.pdf]

Electronic Supplementary Material (ESI) for RSC Advances.
This journal is © The Royal Society of Chemistry 2024

Supporting Information

Tuning Thermal and Graphitization Behaviors of Lignin via Complexation with Transition Metal Ions for the Synthesis of Multilayer Graphene-based Materials

Qiangu Yan [a], Hanwen Zhang [b], Timothy Ketelboeter [a], Yucheng Peng [c, *], Caixia Wan [b, *], Zhiyong Cai [a, *]

[a] Forest Products Lab, USDA Forest Service, One Gifford Pinchot Drive, Madison, WI 53726

[b] Department of Chemical and Biomedical Engineering, University of Missouri, 1406 East Rollins Street, Columbia, MO 65211

[c] College of Forestry, Wildlife and Environment, Auburn University, 602 Duncan Dr, Auburn, AL 36849

* Corresponding authors. Tel: +1 334 844-1089, Email: yzp0027@auburn.edu (Yucheng Peng); Tel: +1 573 884 7882, Email: wanca@missouri.edu (Caixia Wan); Tel: 608-231-9446, Email: zhiyong.cai@usda.gov (Zhiyong Cai).

Number of pages: 4

Number of figures: 3

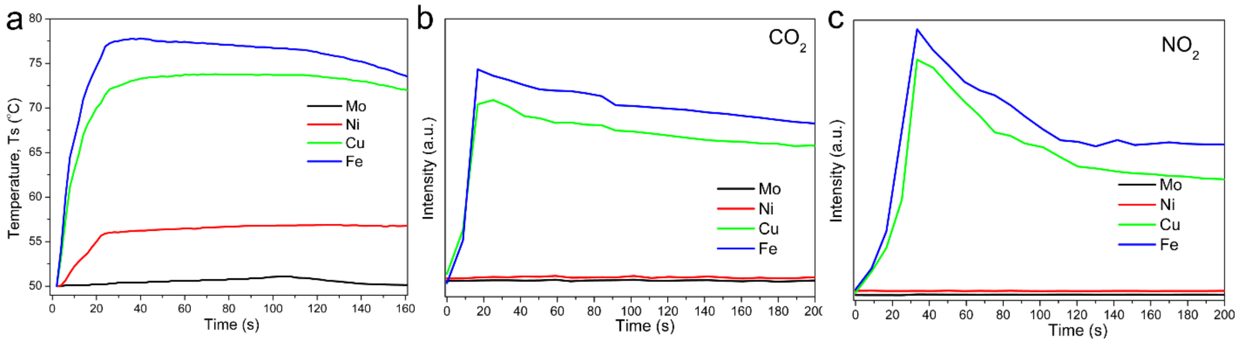

Figure S1. Temperature profiles and gaseous byproducts generated during the M-Lignin complex preparation processes.

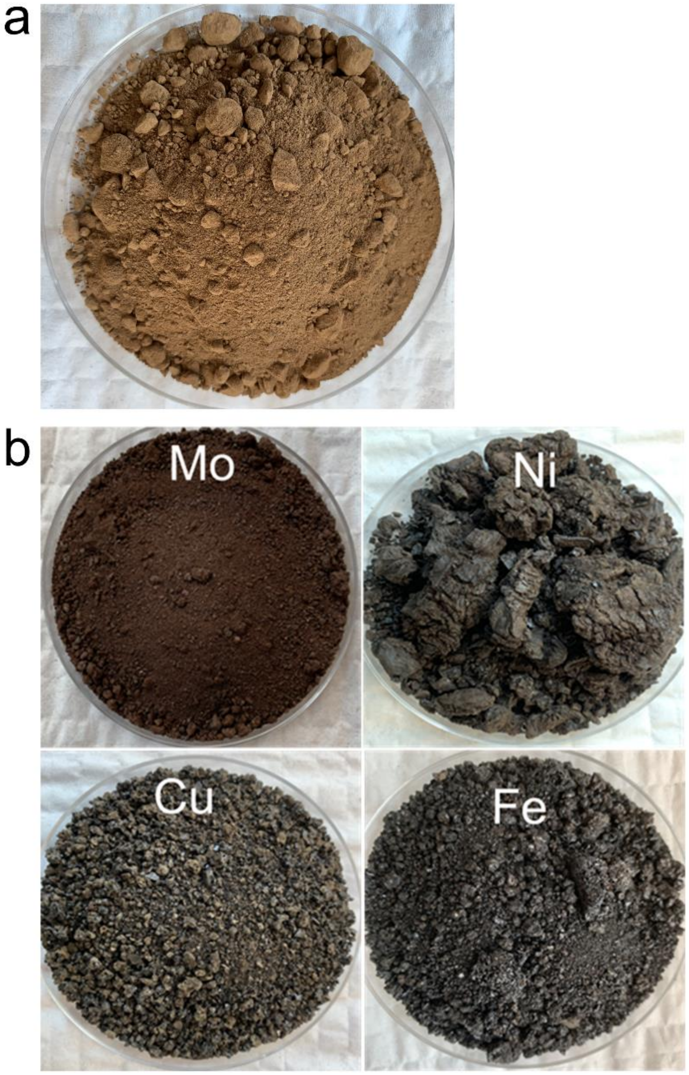

Figure S2. Photos of (a) kraft lignin and (b) M-Lignin complexes.

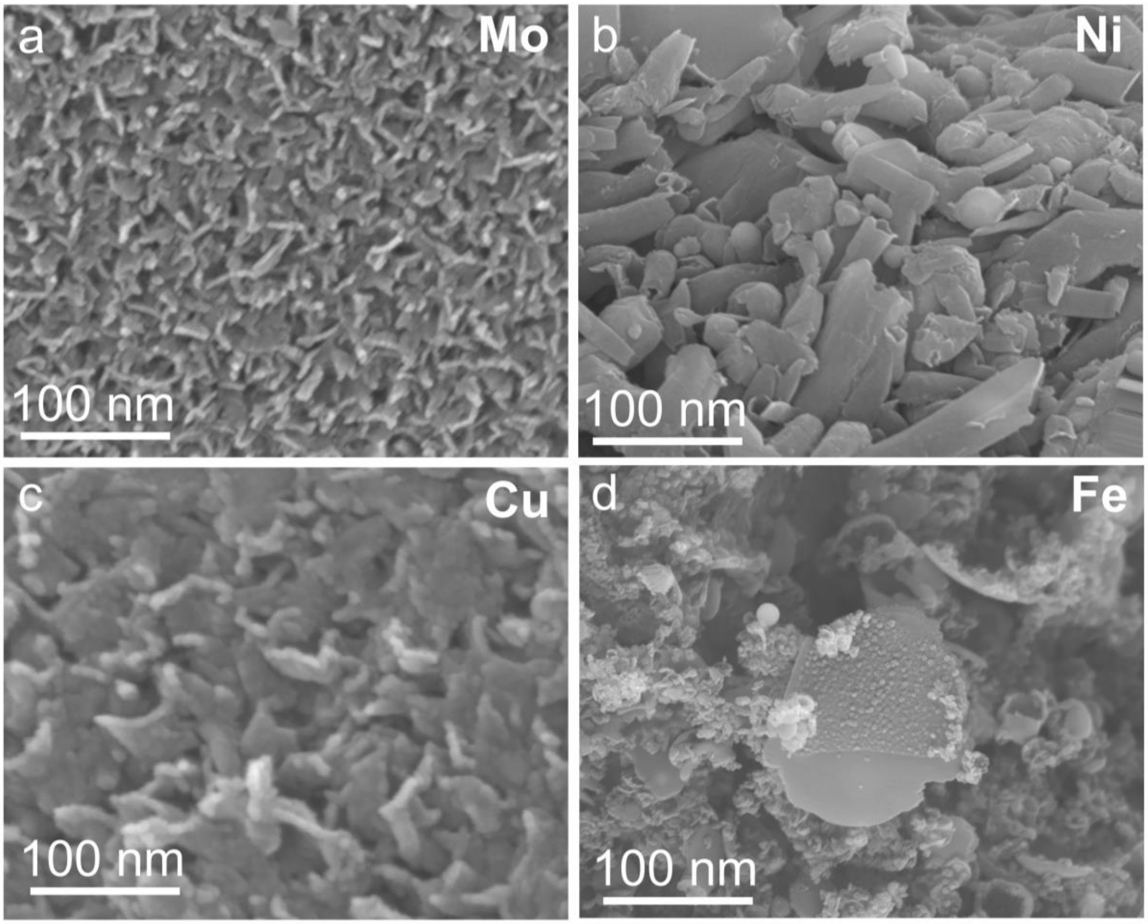

Figure S3. SEM images of microstructure of the products resulting from MCW.
